# Supplementary material for: Clinical and Genetic Characteristics of COL2A1-Associated Skeletal Dysplasias in 60 Russian Patients: Part I
Source: Genes (Basel). 2022 Jan 13;13(1):137. doi: 10.3390/genes13010137 (PMC8775336; doi:10.3390/genes13010137)
Supplement: Supplementary file 1 [file genes-13-00137-s001.zip › Tables with mutations in COL2A1 gene 2.pdf]

TableS1. Supplementary materials. Distribution of variants and characteristics of skeletal and extra-skeletal features in probands

Patient №	Sex	Age y.o	SDS height	Nucleotide substitution	Amino Acid substitution	Family cases	Platyspondyly	Coxa vara	Myopia	Cleft palate	SNHL	Phenotype
1	f	5	-0,71	c.620G>T	p.Gly207Val	+	-	-	-	-	-	Mild SED
2	f	12	-3,7	c.654+4dupA		-	+	-	+	+	-	STL1
3	f	4	-4,65	c.709-1G>T		-	+	+	+	-	+	KD
4	f	13	-0,17	c.817-1G>A		-	-	-	-	-	-	SPPD
5	m	5	2,17	c.823C>T	p.Arg275Cys	-	+	-	-	-	-	KD
6	f	2	-3,47	c.905C>T	p.Ala302Val	-	-	+	+	+	+	SEDC/KD
7	f	2,5	-1,38	c.926G>T	p.Gly309Val	+	+	-	-	-	-	Mild SED
8	f	9	-3,42	c.970-8T>G		-	+	+	+	-	+	KD
9	m	13	-6,1	c.980G>T	p.Gly327Val	-	+	-	-	+	-	SEDC
10	f	4	-1,11	c.1023+1G>C		-	+	-	+	+	+	KD
11	f	8	-2,11	c.1043G>T	p.Gly348Val	-	-	-	-	-	-	Mild SED
12	f	4	-5,3	c.1068+1G>C		-	+	+	+	-	+	KD
13	f	5	-2,18	c.1069G>A	p.Gly357Ser	-	-	-	-	-	-	Mild SED
14	f	16	-1,08	c.1090G>T	p.Gly364Cys	+	+	+	-	-	-	SPPD
15	m	6	-5,51	c.1195G>A	p.Gly399Arg	-	+	+	-	+	-	SEDC
16	f	7	-1,36	c.1266+5G>C		-	+	+	+	-	-	KD
17	f	11 months	1,16	c.1348G>C	p.Gly450Arg	-	-	-	+	-	-	SEMD
18	m	8	-3,87	c.1421_1426del	p.Gly474_Pro475del	-	+	+	+	+	+	KD
19	m	14	-7,16	c.1484G>A	p.Gly495Glu	+	+	+	+	+	+	SEMD
20	f	7	-2,91	c.1510G>A	p.Gly504Ser	-	+	-	-	-	-	SEDC
21	f	17	-4,90	c.1510G>A	p.Gly504Ser	+	+	+	-	-	-	SEDC
22	f	2	-2,17	c.1636G>A	p.Gly546Ser	-	-	-	-	-	-	Mild SED
23	f	7	-4,80	c.1636G>A	p.Gly546Ser	-	+	+	-	-	-	SEMD
24	f	11	-4,40	c.1681G>A	p.Gly561Ser	-	+	+	-	-	-	SEDC
25	f	3	-2,93	c.1780G>A	p.Gly594Arg	-	-	-	+	-	-	SEDC
26	f	17	2,2	c.1833+1G>A		-	-	-	+	+	-	STL1
27	f	7	-9,70	c.2005G>A	p.Gly669Ser	-	+	-	+	-	-	SEDC
28	m	8	-2,21	c.2059G>A	p.Gly687Ser	-	-	+	-	-	-	Mild SED
29	m	8	-7,51	c.2095G>T	p.Gly699Cys	-	+	+	+	-	-	SEDC
30	m	10	0,96	c.2382delT	p.Gly795fs	+	-	-	+	-	-	STL1
31	f	6 months	-5,65	c.2600G>A	p.Gly867Asp	-	+	+	-	-	-	SEDC
32	m	6	-1,95	c.2600G>T	p.Gly867Val	-	+	-	-	-	-	Mild SED
33	f	12	-5,00	c.2609G>A	p.Gly870Glu	+	+	+	-	-	-	SEMD
34	m	9 months	-5,32	c.2617G>A	p.Gly873Arg	+	+	+	-	-	-	SEDC
35	m	6	-7,27	c.2671G>A	p.Gly891Ser	-	+	+	-	-	-	SEDC

36	m	2	-7,41	c.2671G>A	p.Gly891Ser	-	+	+	+	-	-	SEDC
37	f	9	-6,01	c.2671G>A	p.Gly891Ser	-	+	+	+	-	-	SEDC
38	f	12	-1,85	c.2710C>T	p.Arg904Cys	-	-	-	+	+	+	STL1
39	m	10	-1,51	c.2710C>T	p.Arg904Cys	-	-	-	+	-	+	STL1
40	m	6	0,24	c.2813dupC	p.Gly939Trpfs*5	+	-	-	+	+	-	STL1
41	m	11	-1,3	c.2833G>A	p.Gly945Ser	-	+	-	-	-	-	Mild SED
42	f	1 month	1,61	c.2839C>T	p.Gln947Ter	+	-	-	+	+	-	STL1
43	f	4	-8,00	c.2974A>G	p.Arg992Gly	-	+	-	+	-	-	SEDC
44	f	7	-8,73	c.3121G>A	p.Gly1041Ser	-	+	+	-	-	-	SEMD
45	f	1	-3,89	c.3346G>T	p.Gly1116Cys	-	+	+	-	-	-	SEDC
46	m	9	-0,57	c.3397C>T	p.Arg1133Cys	+	-	-	-	-	-	Mild SED
47	f	5	-4,2	c.3442_3444del TCT	p.Ser1148del	-	+	+	+	-	-	SEDC/KD
48	f	5	-5,29	c.3463G>C	p.Gly1155Arg	-	+	+	-	-	-	SEDC
49	f	14	-6,99	c.3464G>T	p.Gly1155Val	-	+	+	-	-	-	SEDC
50	f	9 months	-4,44	c.3464G>T	p.Gly1155Val	-	+	+	-	+	-	SEDC
51	f	11 months	-3,65	c. 3554G>A	p.Gly1185Glu	-	+	+	-	-	-	SEMD
52	m	3 months	-4,58	c.3589G>C	p.Gly1197Arg	-	-	-	-	+	-	SEDC
53	f	7	-7,8	c.3589G>A	p.Gly1197Ser	-	+	+	-	-	+	SEDC
54	f	4	-4,86	c.3627_3644del	p.Pro1211_Pro1216del	-	+	+	-	-	+	SEDC/KD
55	m	11	-3,15	c.3713A>G	p.Tyr1238Cys	-	+	-	+	-	+	STL1
56	m	8	-0,8	c.3897G>T	p.Trp1299Cys	-	+	+	-	-	-	Mild SED
57	f	8	-2,5	c.3950T>G	p.Met1317Arg	+	-	-	-	-	-	Mild SED
58	m	12	5,4	c.4074+1G>A		-	-	-	+	-	+	STL1
59	m	11	-1,8	c.4133T>A	p.Leu1378Gln	-	-	+	-	-	-	Mild SED
60	m	14	3,1	c.4317+1G>T		-	-	-	+	-	-	STL1

Table S2. Supplementary materials. Description of novel variants identified in the sample

№	Nucleotide substitution	Amino Acid substitution	Number of patients with novel confirmed mutation	Clinical significance ACMG Guidelines, 2015	ClinVar ID	Phenotype
1	c.620G>T	p.Gly207Val	1	Pathogenic	SCV002029090	Mild SED
2	c.654+4dupA		1	Likely pathogenic	SCV002029089	STL1
3	c.817-1G>A		1	Pathogenic	SCV002029114	SPPD
4	c.970-8T>G		1	Likely pathogenic	SCV002029091	KD
5	c.980G>T	p.Gly327Val	1	Pathogenic	SCV002029092	SEDC

6	c.1023+1G>C		1	Pathogenic	SCV002029113	KD
7	c.1068+1G>C		1	Pathogenic	SCV002029116	KD
8	c.1069G>A	p.Gly357Ser	1	Pathogenic	SCV002029093	Mild SED
9	c.1090G>T	p.Gly364Cys	1	Likely pathogenic	SCV002029094	SPPD
10	c.1195G>A	p.Gly399Arg	1	Pathogenic	SCV002029095	SEDC
11	c.1266+5G>C		1	Likely pathogenic	SCV002029096	KD
12	c.1348G>C	p.Gly450Arg	1	Pathogenic	SCV002029097	SEMD
13	c.1421_1426del	p.Gly474_Pro475del	1	Pathogenic	SCV002029098	KD
14	c.1780G>A	p.Gly594Arg	1	Pathogenic	SCV002029099	SEDC
15	c.2005G>A	p.Gly669Ser	1	Pathogenic	SCV002029112	SEDC
16	c.2095G>T	p.Gly699Cys	1	Likely pathogenic	SCV002029100	SEDC
17	c.2600G>T	p.Gly867Val	1	Pathogenic	SCV002029101	Mild SED
18	c.2671G>A	p.Gly891Ser	3	Pathogenic	SCV002029102	SEDC
19	c.3346G>T	p.Gly1116Cys	1	Pathogenic	SCV002029103	SEDC
20	c.3442_3444delTCT	p.Ser1148del	1	Pathogenic	SCV002029104	SEDC/KD
21	c.3463G>C	p.Gly1155Arg	1	Pathogenic	SCV002029105	SEDC
22	c.3554G>A	p.Gly1185Glu	1	Pathogenic	SCV002029106	SEMD
23	c.3589G>C	p.Gly1197Arg	1	Pathogenic	SCV002029107	SEDC
24	c.3897G>T	p.Trp1299Cys	1	Pathogenic	SCV002029108	Mild SED
25	c.3950T>G	p.Met1317Arg	1	Pathogenic	SCV002029109	Mild SED
26	c.4074+1G>A		1	Pathogenic	SCV002029115	STL1
27	c.4133T>A	p.Leu1378Gln	1	Pathogenic	SCV002029110	Mild SED
28	c.4317+1G>T		1	Pathogenic	SCV002029111	STL1
